# Signal Processing Techniques for 6G

**DOI:** 10.1007/s11265-022-01827-7

**Published:** 2023-02-02

**Authors:** Lorenzo Mucchi, Shahriar Shahabuddin, Mahmoud A. M. Albreem, Saeed Abdallah, Stefano Caputo, Erdal Panayirci, Markku Juntti

**Affiliations:** 1 Dept. of Information Engineering, University of Florence, Florence, 50139 Italy; 2 Mobile Networks, Nokia, Dallas, 75019 TX USA; 3 Dept. of Electrical Engineering, University of Sharjah, Sharjah, 27272 UAE; 4 Dept. of Electrical and Electronics Engineering, Kadir Has Univ., Istanbul, 34083 Turkey; 5 Centre for Wireless Communications, University of Oulu, Oulu, 90014 Finland

**Keywords:** 6G, Signal processing, MIMO, Optical wireless communications, Internet of bio nano things

## Abstract

6G networks have the burden to provide not only higher performance compared to 5G, but also to enable new service domains as well as to open the door over a new paradigm of mobile communication. This paper presents an overview on the role and key challenges of signal processing (SP) in future 6G systems and networks from the conditioning of the signal at transmission to MIMO precoding and detection, from channel coding to channel estimation, from multicarrier and non-orthogonal multiple access (NOMA) to optical wireless communications and physical layer security (PLS). We describe also the core future research challenges on technologies including machine learning based 6G design, integrated communications and sensing (ISAC), and the internet of bio-nano-things.

## Introduction

Wireless communications networks have evolved to pervasive and ubiquitous enablers for modern societies. The first generation (1G) networks were introduced in 1980s and since then a new generation has emerged every ten years. Each generation from the analog 1G to the current 5G has provided new service features via new technology enablers. The service and quality metrics have focused on improving data rate, reliability, quality, security, and more. While 5G has introduced some initial versions of distributed or edge intelligence to the system design, their actual breakthrough is expected with 6G systems. The technology for 6G networks is now under intensive research and the networks are expected be enrolled in 2030s.

The global data traffic is foreseen to be more than 5000 EB/month in 2030. The value may even increase due to the increasing worldwide use of remote digital services driven by the Covid-19 pandemic. The future society will require fully automated and connected systems. Those will use distributed artificial intelligence (AI) and machine learning (ML), ultra-dense sensors, fast computing, fully integrated heterogeneous connectivity, etc. Those services will consume huge amounts of data, which needs to be transferred and to large extent to or from mobile sources. Emerging Internet of Everything (IoE) applications will require the convergence of communications, sensing, control, and computing functionalities. Some attempts toward this direction have been made in 5G networks already. The stronger demand of high data rates with low latency and low energy consumption, will require the use of THz spectrum in the radio frequency (RF) domain as well as the use of optical wireless communications (OWC), and in particular of visible light communications (VLC).

Although 5G has started the journey to address the challenges described above, many open problems remain. We need to investigate, e.g., the following: 1) How to utilize higher frequencies with larger bandwidths and lower power communications? 2) How to satisfy the new requirements: ultra low latency and ultra reliable communications? 3) How to guarantee security, also in low-resourced (but fully connected) devices? 4) How to integrate heterogeneous technologies (e.g., radio-optical)? 5) How to use ML/AI and deep learning (DL) for networks and over networks? 6) How to guarantee the power/energy efficiency and material sustainability of the new networks and user devices? 7) How to design the systems and business environment so that it is profitable while the services and applications serve the green transition and environmental sustainability?

The future 6G networks will be designed so that users’ intelligence and needs will be further explored and satisfied. In other words, the various applications of run by the users will form the 6G network operating as a networked computer or inference machine. This calls for power and energy efficient, reliable wireless connectivity and networks.

**Figure 1:**
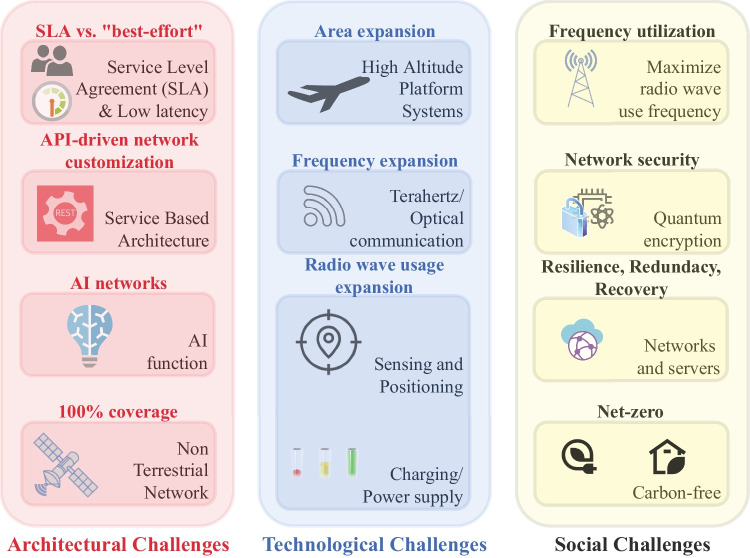
6G challenges.

Potential use cases and challenges (Fig. 1) for 6G connectivity could span from extended reality (enhanced virtual/augmented reality) to interactive robotics and AI-based autonomous systems, from wireless brain-computer interaction to haptic communications, from in-body communications to human-bond communications, from massive Internet of everything to umanned mobility.

### Main 6G System Technologies and Architectures

Since 6G research and projects emerged, papers started to appear in scientific literature, in particular overview papers. In [1], a survey of 6G from the point of view of energy consumption and green architectures and technologies is reported. The first 6G technology white paper was published in 2019 by the Finnish 6G Flagship Program [2]. In [3], a general survey over the technologies envisioned for 6G networks and services is described. A holistic and forward-looking vision that defines the tenets of a 6G system can be found in [4]. In [5], the role of AI in designing and optimizing 6G architectures, protocols, and operations is analyzed. In [6], the potential use cases enable by new technologies of 6G systems are taken into account, while [7] focused on 6G architecture, describing the usage scenarios and requirements for multi-terabyte per second and intelligent 6G networks. The role of deep learning to enhance 6G networks is discussed in [8]. In [9], signal processing is indicated as important factor for the integration of different frequency bands and dynamic resource management. In [10], the use of optical signal processing together with AI is envisioned to revolutionize the next generation mobile networks, while [11] envisions new signal processing methods as mandatory for dealing with future massime MIMO networks as well as for security in 6G networks. In [12], the signal processing is seen as fundamental, together with AI, for network orchestration of future intelligent IoT networks. In [13], the role of signal processing in the 6G era, along with the associated challenges, is briefly discussed, although this is not the main goal of the paper.

While 6G architectural/technological/societal challenges are reported in Fig. 1, core technologies can be classified as

- **Wireless communication systems**: THz communications; visible light communications; nanoscale communications;
- **Next generation antenna and materials**: massive multiple antennas; cell-free MIMO; reflecting intelligent surfaces; radio-reconfigurable antennas;
- **Coding and modulation**: channel coding; non-orthogonal wave; multiple access systems;
- **Spectrum sharing**: free duplex; full duplex; dynamic spectrum sharing;
- **Full integration of**: artificial intelligence; Internet of things; blockchain.

From architectures point of view, the 6G era will show several novelties

- **Ubiquitous 3D coverage**: non-terrestrial networks (NTN); high amplitude platform systems (HAPS); integration of space, aerial, terrestrial, and underwater networks;
- **Intelligence inside networks and systems**: AI-based networking; real-time intelligence (edge computing); intelligent spectrum adaptation;
- **New network protocols**: next Internet protocols.

**Figure 2:**
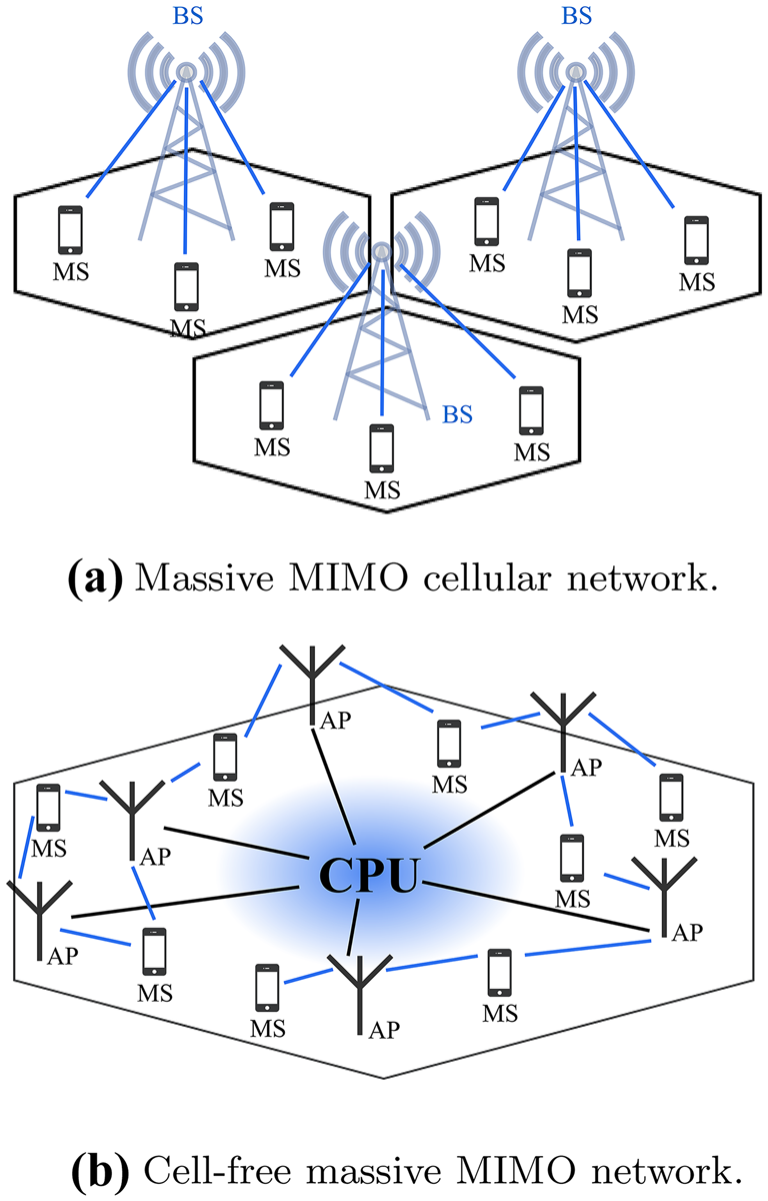
Massive MIMO networks.

The current 5G networks already increase the data rate and decrease the data connections latency. Further performance gains are expected from *cell-free* massive MIMO illustrated in Fig. 2. Massive MIMO is a key enabler already in the 5G networks, while the cell-free extension is largely expected to be introduced to practical use in the 6G era. In cell-free massive MIMO, all multiple-antennas access points (AP)s are connected to a CPU which operates all as a massive MIMO network with no cell boundaries where all users are served by coherent transmission and reception [14]. The APs and the CPU exchange the users’ estimated channels between them, hence, the burden on the fronthaul network is increased providing opportunities for performance enhancements. Cell-free massive MIMO is first introduced by Yang in [15]. It implies that there are no boundaries between cells. Cell-free massive MIMO is a combination of three concepts: massive MIMO, distributed MIMO, and cell without boundaries [14].

### Paper Outline

Although the general literature on 6G is getting rich, very few overview papers on signal processing aspects for 6G have appeared. This paper aims at discussing the role and key challenges of signal processing in future 6G systems and networks. In [8] the signal processing for 6G systems is touched but its main interest is towards the deep learning techniques. Conversely, we discuss the whole signal processing chain in future 6G systems, from the conditioning of the signal at transmission to MIMO detection, from channel coding to channel estimation, from multicarrier (radio) modulation to optical wireless communications.

The paper is organized as follows. We cover frontend signal processing, transmit precoding and beamforming, multiantenna detection, channel coding and decoding, channel estimation, as well as non-orthogonal multiple access (NOMA) signal processing, optical wireless communications (OWC), and physical layer security (PLS) in Sections 2–9. In Section 10, the future research challenges on technologies are discussed. Those include machine learning based 6G design, integrated communications and sensing (ISAC), and the internet of bio-nano-things. Paper is briefly summarized and conclusions are drawn in Section 11.

## Front-End Signal Processing and Digital Pre-distortion

**Figure 3:**
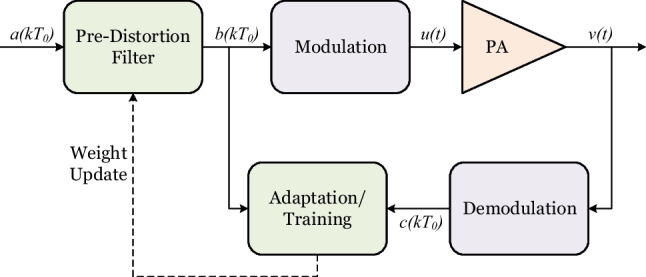
A communication system with DPD.

The non-linear distortion of radio frequency components can severely degrade the performance of an entire communication system. The primary source of the non-linear distortion in radios are typically the high-power amplifier (PA). The non-linearity problem can be circumvented by using linear class A PAs or operating any PA far from their saturation point. However, this leads to a bulky, expensive and inefficient PA which is far from an ideal solution to non-linear distortion. A popular solution is to apply a non-linear filter at the digital domain of the transmitter that applies an inverse of the PA response. This technique to linearize PA response by pre-distorting the digital signal is known as digital pre-distortion or DPD. In Fig. 3, a communication system in the presense of a DPD is illustrated. Here, the baseband signal is denoted by $a(KT_0)$ which traverses through a pre-distortion filter which applies an inverse of the PA response. The coefficients of the pre-distortion filter gets repeatedly updated by the adaptation or training block. The adaptation block compares the output of the PA at the baseband, $c(KT_0)$ and the output of the pre-distortion filter, $b(KT_0)$ to generate the updated coefficients.

DPD for dual-band PAs is a common feature for 5G radios these days. It is an alternative to ultra-wideband DPDs by only compensating for the nonlinear distortions around the signal bands of interest. The dual-band DPD is derived from the wideband memory polynomial DPD model [16]. The individual terms located around each band can be grouped to define the coefficient mapping between the wideband model and its corresponding dual-band model. We envision that the trend to develop more sophisticated DPDs for multi-band will continue throughout the decade. To be more specific, the tri-band DPD products will be more popular by the time 6G arrives. For dual-band case, the terms centered around the signal band are sufficient because the out-of-band intermodulations are far from the band of interest. However, the out-of-band intermodulation terms might be located around the band of interest in concurrent tri-band PAs due to their high volume. Some literature already exists on tri-band DPDs. In [17], the authors presented a DPD for concurrent tri-band PAs. The PA model is based on a pruned Volterra model which takes both phase distortion in multiband PAs and compound amplitude distortion. Conventional least-square (LS) has been used to estimate model coefficients in [17]. A simplified dynamic deviation reduction model and LS is used for modeling the PAs and estimating the model coefficients, respectively, for a tri-band DPD.

The cell-free massive MIMO system requires relatively smaller antennas and analog circuitry for radio-frequency (RF) modules so that the access point (AP) could be placed in any geographical location. This poses an interesting challenge to DPD implementation because the pre-distortion filter is typically placed closer to the RF circuits. Due to the small size of the AP hardware unit close to the user, it might be challenging to implement complex algorithms for the DPD. As the AP distance from the central processing unit (CPU) will be different, it will be challenging to synchronize different feedback signals with a single feedback path. Due to the size of the APs and challenges with the feedback, a non-adaptive simpler DPD solution will be ideal for a cell-free massive MIMO system. A simple look-up table (LUT) based DPD can be used in this case which is typically used for mobile phones. In [18], one such classical LUT DPD has been presented. The AM-AM and AM-PM non-linearities are characterized to calculate complex coefficients which are placed in the LUT.

6G communication systems will use very high carrier frequencies and thus, beamforming is essential to circumvent the high attenuation and path loss. Phased array based beamforming transmitters typically apply multiple PAs for a single digital stream. However, this introduces significant challenges for DPD because multiple parallel PAs have to be linearized with a single DPD. According to [19], the two methods to design such a DPD would be to linearize individual PA as a LS problem or to linearize array response to a desired spatial direction. In [20], the authors presented a DPD scheme that can linearize multiple PAs of a hybrid system with antenna subarrays. The authors used LS to identify the PA parameters. The identified parameters and the input signal were used to design the DPD block which minimizes the expected sum of squared error. To accelerate the convergence, the least mean-square (LMS)-Newton algorithm was used for iteratively minimizing the expected sum error. However, this scheme is less effective because the errors are measured independently and they can add up constructively or destructively over the air. Therefore, minimizing the array error in the desired beam direction provides better results. In [21], the authors formed the cost function with the theoretical nonlinear behavior in the array far-field and input signal, which was solved by LS.

The PA models are generally not perfect as the response of a PA is a non-linear continuous function. The PA has to support different types of signals and thus, the PA modeling can be challenging for a rapidly changing environment. Neural networks can be very efficient for non-linear mappings. A feedforward neural network with sufficient neurons is known as universal approximators for an arbitrary continuous function [22]. Therefore, the neural network has been a popular choice for DPD modeling. For example, in [23], the authors proposed a neural network DPD model to jointly mitigate crosstalk, I/Q imbalance and nonlinearity in MIMO transmitters. During the feedforward computation, the authors initially set the weights between $[-0.8,0.8]$ and during backward propagation weights are adjusted to reduce the error. The Levenberg-Marquardt algorithm, which is an approximation of the Gauss-Newton method, is used for updating the coefficients. We expect deep neural networks to play an important role for 6G DPD modeling.

## Transmit Precoding and Beamforming

In a wireless propagation environment, it is usually hard to obtain a reliable channel state information (CSI) where the performance of a downlink (DL) transmission system could largely be affected. Precoding algorithms can be exploited to deal with imperfect CSI where the effects of interference and path-loss are reduced. Precoding can also be exploited at the MIMO’s base station (BS) to increase the spectral efficiency, and enhance the throughput and the capacity when the number of antennas approaches infinity [24]. In literature, linear, non-linear, and machine learning based precoding algorithms were proposed. Usually linear precoding techniques, such as the zero-forcing (ZF) and the MMSE, depend on multiplying the transmitted signal by the precoding matrix [25]. However, such methods incur a high complexity due to a matrix inversion. Linear precoders based on approximate matrix inversion methods such as the Neumann series approximation (NSA), Newton iteration (NI), Chebyshev iteration (CI), Gauss-Seidel (GS), conjugate gradient (CG) and successive overrelaxation (SOR), were proposed. Although approximate matrix inversion methods achieve a satisfactory performance when the ratio between the number of BS antennas and user terminals is large, they have a severe performance degradation when the ratio is small. They also need to calculate an initial value which could slow the convergence. In addition, many methods need additional calculations to find suitable relaxation/optimization factors. Matrix decomposition algorithms provide better numerical stability over approximate matrix inversion methods at the cost of a high computational complexity [26]. However, semiconductor technology has also matured greatly over the last ten years where the interest has been shifted towards better system design than saving logic area within unstable and risky solutions. Several precoders have been proposed based on QR and Cholesky decompositions [26].

The second class of precoders is the non-linear such as the dirty-paper-coding (DPC) [27], Tomlinson-Harashima (TH) [28], and vector perturbation (VP) [29] based precoders. The DPC algorithm is not hardware friendly because infinite length of codewords and sophisticated signal processing are required. The TH precoder is a suboptimal implementation of the DPC algorithm and decomposition. In comparison with the DPC based precoders, TH precoder is more hardware friendly. The generalized TH algorithm, also known as VP algorithm, obtains a much lower complexity compared with the DPC algorithm. In the VP algorithm, the data is aligned to the eigenvalues of the channel matrix inverse on an instantaneous basis. It performs a sphere search out of several candidate perturbation vectors to reduce the precoded signal norm [29].

In order to improve the achievable downlink data rates of a cell-free massive MIMO, conjugate beamforming (CB) precoding [30], ZF precoding [31] and MMSE precoding [32] have been utilized. It is shown that the centralized MMSE significantly achieves higher DL data rates compared to conventional CB precoding. In centralized approach, the APs and the CPU exchange the users’ estimated channels between them, hence, the burden on the fronthaul network is increased. Therefore, several advanced local precoding techniques are proposed to eliminate such burden such as the local full-point ZF (FZF) [33], partial ZF (PZF), and protective partial ZF (PPZF) [34].

**Figure 4:**
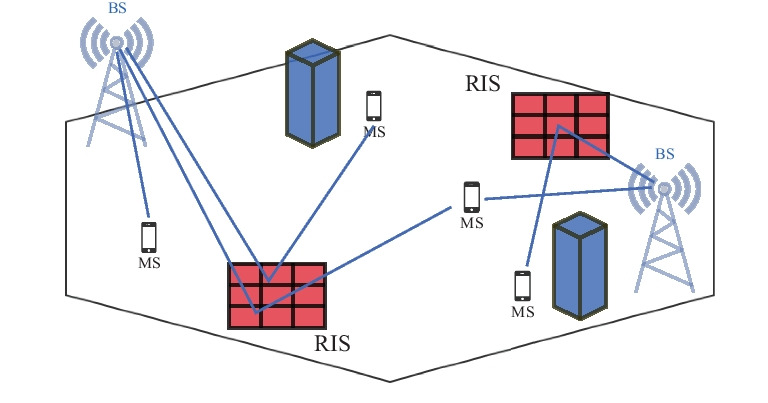
Reconfigurable intelligent surfaces.

An important recent extension of the conventional beamforming or precoding is presented by the *reconfigurable intelligent surface* (RIS) technology, known also as intelligent reflecting surface (IRS) [35–38] illustrated in Fig. 4). A RIS typically consists of a large number of low-cost passive elements, and can support different functional modes, e.g., reflection, polarization, refraction, and absorption. A RIS can be constructed, e.g., by varactor diodes or crystal liquid [39, 40]. A ray tracing based RIS channel model was proposed in [41] for both indoor and outdoor environments. The objective of the RIS is to control the wireless propagation environment so as to enable improved connectivity [35, 42, 43]. What is more, the RIS can also be considered for localization together with mmWave communications technology networks [44–46].

One key limitation of the passive RIS is the fact that the passive beamforming limits the beamforming gains or the available degrees of freedom. Large numbers of RIS elements are needed to outperform the decode-and-forward (DF) half duplex relaying with moderate numbers of antennas [47, 48]. What is more, a practical RIS often applies limited-resolution phase shifters resulting in further performance penalty [49, 50]. Therefore, the RIS performance can be improved by so called *hybrid relay-reflecting* (HR-RIS), which combines the functionalities of a RIS and a relay [51]. The basic idea of the HR-RIS is to replace few elements of the RIS by simple reflect amplifiers.

## MIMO Detection

**Figure 5:**
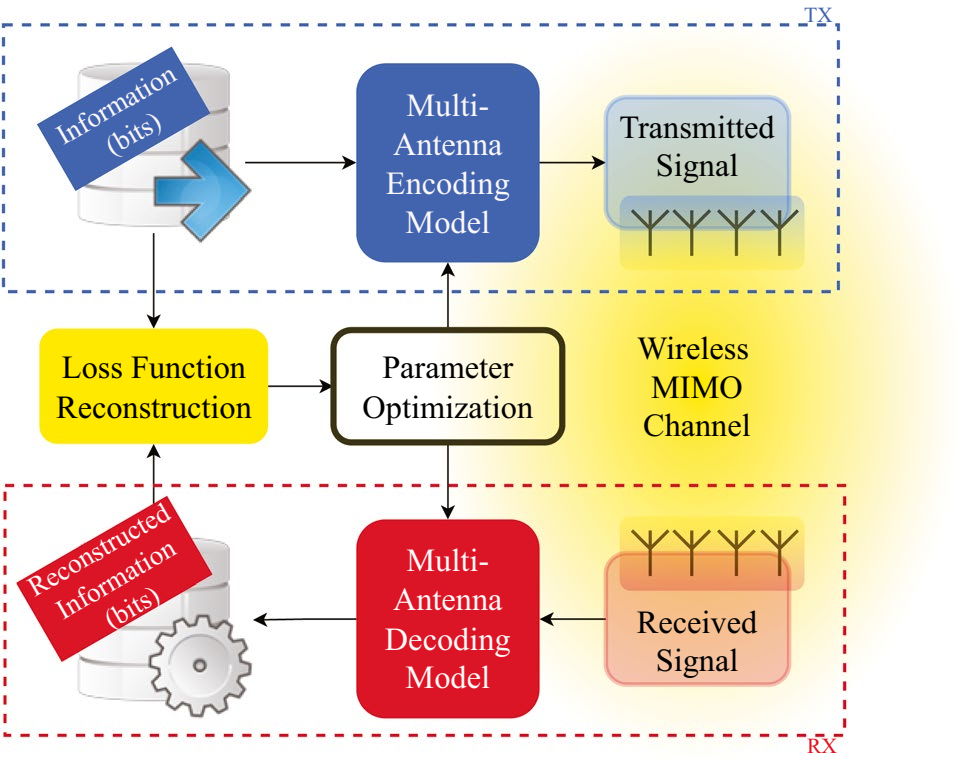
SP MIMO processing scheme.

In the last few years, there is a debate to choose the most scalable massive MIMO scheme: centralized vs. decentralized. In centralized massive MIMO, the central processing unit (CPU) collects the CSI from all antenna elements. The signal processing tasks (demodulation, decoding, etc) are performed at the CPU which require extra radio frequency (RF) and analog-to-digital converter (ADC) components (Fig. 5). In the last few years, cell-free massive MIMO has gained a lot of attention due to its potential to improve the energy and spectral efficiencies of wireless communication systems. In cell-free massive MIMO, data detection is performed locally at each AP, centrally at the CPU, or partially first at each AP and then at the CPU. Most of the detection techniques for cell-free massive MIMO are centralized as they rely on a single CPU to do most digital signal processing (DSP) tasks with the help of irregular distributed APs.

The first centralized massive MIMO detector has used the likelihood ascent search (LAS) because of its linear average per bit complexity in number of users and its ability to achieve a near-maximum likelihood performance. Unfortunately, the bit-error-rate (BER) is significantly deteriorated in the scenario of high modulation order and realistic environment. In addition, a computation of the initial vector includes a matrix inversion which increases the computational complexity. Reactive tabu search (RTS) is another local search method where more restrictions are introduced to avoid an early termination, and hence, higher accuracy is achieved. Unfortunately, the RTS detector suffers from a high computational complexity and a performance loss when a high modulation order is used. In order to reduce the complexity, most of the proposed detectors during 2008 - 2013 had used local search algorithms and belief propagation (BP) algorithms. The BP algorithms, such as the message passing and the Bayesian belief networks, iteratively search for the optimum solution in a space where the damping factor (DF) is carefully optimized. The BP is very sensitive to both the message update rules and prior information. However, it achieves a high performance when the correlation between the channel elements is relatively small. In years after, due to fail to guarantee convergence and implementation difficulties, a research on linear and nonlinear detectors based on free-matrix-inverse methods has been conducted. Unfortunately, these detectors suffer from a high performance loss and a high computational complexity when the massive MIMO size is large, the ratio between the BS antennas and user antennas is small, and an existence of a high correlation between channel elements. Researchers in the telecommunication industry intend to improve the system design to avoid unstable and risky solutions for their products. Therefore, in [52], we present the computational complexity of linear detection mechanisms based on the QR, Cholesky and LDL decomposition algorithms for different massive MIMO configurations. Other detectors, such as the sphere decoding (SD), suffer from a high computational complexity. Therefore, most existing detectors need a refinement to meet the implementation demands of a low computational complexity and high performance, in particular under complicated environments.

Since 2017, there is a substantial trend in the research community to exploit machine learning, artificial intelligence (AI), and deep learning (DL) in data detection. The deep network in the massive MIMO detector’s design based on projected gradient descent method is utilized and called DetNet [53]. It performs well in i.i.d. Gaussian channel and low-order modulation schemes (i.e., BPSK and 4-QAM). A modified DetNet [54] is proposed where a relatively small number of parameters is required to optimize. Unfortunately, the training is unstable in realistic and correlated channels. In addition, scalability of the DetNet algorithm is poor because of a relatively large number of training parameters. In 2018-2022, there is a notable trend in a research community to exploit the DL to build a robust massive MIMO detector. A model-driven DL network is proposed based on the orthogonal approximate message passing network (OAMP-Net) [53]. It adds some adjustable parameters to the existing OAMP method. Unfortunately, it is very restrictive where a strict assumption has to exist. The performance of the OAMP-Net is dominated by the matrix inverse in each layer. Therefore, it is not feasible for implementation because of a high complexity. OAMP-Net2 algorithm is an extension of the OAMP-Net where new training parameters are utilized [53]. Unlike the OAMP-Net, imperfect channels are considered. However, like the OAMP-Net, it is dominated by the matrix inverse. The MMNet [55] algorithm is proposed to overcome the challenges in the DetNet and the OAMPNet. It is designed to be trained online for each where an iterative soft thresholding algorithm is used. Although it achieves a good performance when implemented in a realistic channel simulator, it incurs latency due to the sequential online training. In addition, the performance degrades significantly in a high modulation order. A HyperMIMO [56] based detector replaces the training process required by the MMNet for each channel realization by a single inference through a trained hyper-network. It also reduces the number of parameters of the MMNet. In comparison with the MMNet, HyperMIMO performs slightly worse. It also needs to be re-trained when the channel statistics change significantly.

Many testbeds, such as the Argos testbed, the LuMaMi testbed, and the BigStation testbed, are available to support the decentralized channel estimation and data detection at antenna elements. Unfortunately, they rely on the maximum ratio combining (MRC) that significantly reduces spectral efficiency, and hence, prevents the use of high-rate modulation and coding schemes. Therefore, alternative (BS) architectures based on a decentralized approach are proposed. A decentralized data detection method based on the (CG) is proposed where the BS antenna array is partitioned into clusters and each cluster is associated with independent local (RF) elements and computing circuitry [57]. Another decentralized data detection based on alternating direction method of multipliers (ADMM) [58], partially decentralized (PD) and fully decentralized (FD) data detectors based on approximate message passing (AMP) [59] are proposed. Unfortunately, the proposed decentralized based processing (DBP) is not tested in different system configurations and realistic channel conditions. The DBP is studied based on free-matrix-inversion methods in different channel conditions [60]. The FD [61] architectures based on the coordinate descent (CD) method and FD [61] data detector based on recursive least square (RLS), stochastic gradient descent (SGD), and averaged stochastic GD (ASGD) have also been proposed.

DL architectures and (AI) could be exploited in decentralized and cell-free massive MIMO. However, the literature has shown a paucity of employing artificial intelligence for data detection in decentralized and cell-free massive MIMO.

## Channel Coding

Channel coding is a technique to control errors in data communication over noisy channels. The key idea of channel coding is to add redundancy to the messages in the transmitter for encoding. These redundant parts are used on the receiver side to detect the errors. Channel coding is an integral part of wireless communication systems since the introduction of the convolutional codes in 1955 by Elias [62]. In 5G new radio (NR) standard, low-density parity-check (LDPC) and polar coding are adopted data and control channels, respectively [63]. Therefore, LDPC and polar coding schemes will continue to evolve throughout this decade. We envision that improved versions of LDPC and polar will be major candidates for 6G channel coding. The first reason is we have seen in the past that popular channel coding schemes have been adopted for more than one generation of communication systems. For example, turbo coding has been adopted for both 3G and 4G systems. The second reason is LDPC and polar are already very good channel coding schemes. LDPC are capacity-approaching codes and polar are the first capacity achieving codes with low decoding complexity. The third reason is the existing hardware implementations in the literature can already provide hundreds of Gbps [64, 65]. Therefore, we can assume that with improved algorithm and hardware architectures LDPC or polar schemes will be able to provide Tbps throughput required for 6G communication systems.

LDPC introduces more complexity in the encoding phase, but the decoding phase is simpler than turbo codes and thus, ideal for large block lengths. However, to reach the Tbps goals of 6G, the LDPC algorithms will require further modification and optimization. In [66], the authors proposed a Check Node Self-Update (CNSU) algorithm for LDPC decoding which reduces the memory and power requirements. The hardware architecture based on CNSU algorithm was able to provide very high throughput which makes it suitable for beyond 5G systems. In [67], the authors studied the convolutional code LDPC (CC-LDPC). The authors compared the CC-LDPC to the conventional block code LDPC schemes. CC-LDPC has the advantage in terms of lower error floor, faster decoding convergence and lower decoding complexity. The authors concluded that CC-LDPC has enormous potential for 6G communications due to its high reliability and low latency. There have been reinvigorated interest in protograph-based LDPC as protographs provide an efficient way to construct LDPC codes. In [68], the authors proposed an improved protograph LDPC algorithm suitable for 1-bit ADC-based massive MIMO systems. This new method overcomes the error-floor issue of conventional LDPC codes and is a suitable candidate for low resolution 6G base stations. NOMA schemes have great potential to be an integral part of 6G which opens a new area of multi-user oriented channel coding [69]. Interleave-division multiple-access (IDMA), a capacity-approaching NOMA scheme, with turbo joint decoding at the receiver can leave a gap of 1.4 dB to Shannon limit at the sum spectral efficiency with 16 users [70]. Recently, LDPC codes have been proposed for multiuser channels due to their lower complexity and flexibility. For example, a raptor-like quasi-cyclic LDPC has been constructed for IDMA based random access in [71].

Polar codes, which was introduced in 2009, is the first capacity achieving codes with low encoding and decoding complexities. However, sequential coding (SC) is required for the polar coding schemes to reach Tbps throughput. SC decoding traverses through a polar factor tree in a sequential manner which can be unrolled for high throughput [72]. Even though SC decoding enables very high throughput, they suffer from error correcting performance. Successive cancellation list (SCL) processes only a subset of candidates among the polar factor tree nodes. At leaf nodes, the less reliable candidates are sorted out. The sorting process introduces marginal complexity which is negligible due to an improved error correction performance. We would also like to mention that 6G systems might replace LDPC codes and adopt polar codes also for the data channels.

Most channel codes are designed for a specific set of coding rates. Even though LDPC provides a large choice of coding rates for 5G, they are not truly rate-less. In [73], the authors presented a novel rate-less code which they named as *spinal codes*. This novel coding scheme uses a hash function over the message to generate pseudo-random bits which can be mapped directly to the constellations. The simulation results show that spinal codes achieve Shannon capacity and outperform the best-known fixed rate block codes. Therefore, spinal codes will enable a rate-less 6G system where any coding rate can be used for transmission based on the receiver capacity and channel condition.

Deep learning will play a key part in 6G systems. Channel coding methods based on deep learning have also gained a lot of attention in recent years. Deep learning has been used to decode linear codes in [74]. The application of deep learning improved the performance of the belief propagation algorithm. The improvements were demonstrated by different LDPC codes. The polar decoder is enhanced by applying deep learning in [75]. The authors partitioned the encoding graph and train them individually which results in a non-iterative and highly parallel decoder. In [76], a trained deep neural network is concatenated with a standard belief propagation decoder. Iterating between the neural network and belief propagation resulted in better decoding performance.

## Channel Estimation

Channel estimation continues to be an essential receiver functionality in 6G systems. Several of the key technologies envisioned for 6G impose new channel estimation problems that cannot be solved efficiently using conventional methods. For instance, THz communication experiences significantly long channel responses as well as low SNR due to the srong noise [77]. Moreover, minor variations in the environment can cause significant channel estimation errors [78]. Furthermore, massive MIMO systems operating in the THz range experience the beam split effect where the path components split into different spatial directions at different subcarrier frequencies, leading to serious array gain losses [79]. RISs also introduce new challenges in channel estimation since RIS elements are passive and cannot transmit, receive, or process any pilot signals to realize channel estimation. It is therefore important to develop channel estimation algorithms that can handle such challenges. These algorithms should combine low computational complexity with high spectral efficiency (low pilot overhead), without sacrificing the estimation accuracy. In what follows, we will summarize the state of the art in channel estimation for 6G systems.

The enormous bandwidth available for THz communication enables the achievement of data rates in the order of 1 Tbps. The unprecedented potential of THz communication also comes with major practical challenges for implementation, including high propagation losses due to severe signal attenuation and molecular absorption, as well as the frequency selectivity of the channel. Considering the large number of channel parameters and the unsuitability of conventional estimation techniques, most works in the literature either exploit the inherent sparsity characteristics of the channel through compressed sensing (CS), or leverage the power of deep learning to reduce the computational complexity and improve the estimation accuracy and spectral efficiency.

CS was applied to estimate indoor THz channels in [77]. In addition to proposing a compressive sampling matching pursuit (CoSaMP) algorithm, the authors of [77] also considered the Dantzig selector (DS), a computationally tractable CS-based approach that formulates the channel estimation as a convex optimization problem. It was shown that the both CS methods significantly outperform the least squares (LS) approach in terms of MSE, and that the DS method is preferable to the CoSaMP method which exhibits some degree of instability for low number of observations. The work in [80] applies CS to estimate dynamic MIMO THz channels by exploiting sparsity in the angular/delay domain. An algorithm is proposed based on accelerated gradient descent with adaptive restart (AGDAR), which is shown to be fast and effective. Moreover, two further improved CS algorithms are introduced, namely the selective AGDAR (S-AGDAR) and the adaptive AGDAR (A-AGDAR). Substantial gains in MSE, computational complexity and latency are observed over the LS method. A joint activity detection and channel estimation (JADCE) technique is proposed in [81] for wideband THz IoT systems to address the large pilot overhead and the large dimensionality of the signal processing, by exploiting both the sparsity pattern in the angular domain and the low-rank structure of the channel matrix.

While [77] and [81] consider SISO systems, and [80] considers a MIMO system, massive MIMO THz channel estimation is more complicated due to the very large number of channel parameters. Accurate channel estimation is essential to enable hybrid precoding and to reduce the number of RF chains. Furthermore, massive MIMO THz systems experience the beam-split effect, where the large number of antennas and the wide bandwidth result in frequency-dependent sparse channel supports and make the spatial channel directions different from each other in the angular domain for different subcarriers. Channel estimation for this scenario is studied in [82], where beam split patter detection is first performed, and then the sparse channel supports at different subcarriers are estimated using a support detection window. The procedure is repeated until all path components are considered, and the wideband channel is recovered by considering the total sparse channel support containing the channel supports for the different path components.

The work in [83] also addresses the beam split effect in THz massive MIMO systems by using uniform planar arrays. Channel sparsity in the angular domain is exploited to formulate the channel estimation problem as a CS problem, which is solved using the orthogonal matching pursuit (OMP) algorithm. Contrary to the existing works, the authors employ a wideband dictionary and show that the channels across different OFDM subcarriers share a common support in this case. This enables applying a variant of the simultaneous OMP algorithm, coined as generalized simultaneous OMP (GSOMP), which exploits the information of multiple subcarriers to increase the probability of successfully recovering the common support. It is reported that the proposed GSOPM outperforms the OMP in the low and moderate SNR regimes.

Other works have sought to leverage the power of deep learning for channel estimation in THz massive MIMO systems. Considering an array-of-subarrays configuration, [84] develops a deep convolutional neural network (DCNN) channel estimation technique that learns the parameters of the spherical wave channel model, including azimuth and elevation angles, amplitude of the channel gain and phase shift matrix. The work in [85] addresses the inaccuracies of the planar wave model and the limitations of the spherical wave model by proposing a hybrid planar-spherical model. The planar wave model is adopted within sub-arrays and the spherical model among subarrays. A combination of DCNN and geometric relationships is employed to estimate the channel parameters over two stages. The work in [86] exploits both machine learning techniques and the sparsity structure of the channel matrix by designing a hybrid transceiver where estimation is performed via a combination of Bayesian learning and orthogonal matching pursuit (OMP). Generative adversarial networks (GANs) are trained in [87] to generate samples from the unknown channel distribution. The trained network is then used as a prior to estimate the current channel using the received signal.

CSI acquisition for RIS-assisted networks is another challenging problem due to the compound nature of the propagation. However, it is necessary for the RIS phase control, beamforming, resource allocation, and interference management [88]. Several channel estimation algorithms have been proposed. The RIS is often assumed to be used in the mmWave or (sub-)THz communications systems to enable line-of-sight (LOS)-like connectivity even with the non-line-of-sight (NLOS) conditions. The mmWave channels are typically very directive and sparse with a small number of propagation paths. Numerous compressive sensing (CS) based approaches, e.g., the atomic norm minimization, basis pursuit, approximate message passing (AMP), and mixed norm minimization have been proposed for RIS channel estimation [89–94]. Joint channel estimation and data-rate maximization for THz-based RISs is proposed in [95] through an iterative atom pruning based subspace pursuit (IAP-SP) scheme, which is noted to exhibit lower computational complexity than the classical subspace pursuit (SP) scheme. A two-stage algorithm that includes a sparse matrix factorization stage and a matrix completion stage is developed in [96], and a novel message-passing based algorithm is proposed to solve the matrix-calibration based matrix factorization problem in [97].

In [98], the channel is estimated for the downlink MISO RIS system, with the assistance of active elements that are randomly distributed in the RIS, which successfully reduces the pilot overhead. On the other hand, an uplink multi-user MISO RIS system is considered in [99], and the channel is estimated based on parallel factor decomposition to unfold the cascaded channel model. The channel is then estimated using alternating least squares and vector approximate message passing.

Geometric channel models explicitly couple the channel parameters and node locations leading naturally to joint channel estimation and mobile positioning [100]. In addition to conventional model-based approaches, data-driven approaches, for instance, deep learning can also be employed for channel estimation, positioning, RIS phase control, and symbol detection.

Cell-free massive MIMO is another promising 6G technology where channel estimation plays a critical role. Channel estimation enables the evaluation of the precoding/detection vectors used for DL/UL data transmission. An overview of channel estimation techniques for cell-free massive MIMO is provided in [101]. As noted in [101], most techniques are based on pilot transmission where both orthogonal and non-orthogonal pilot schemes have been studied. Orthogonal schemes are more suitable scenarios with low mobility and a small number of users, whereas non-orthogonal schemes are preferable for high mobility scenarios. Most of the works adopt the minimum-mean squared error (MMSE) estimation scheme, and significant effort has been expended to limit the impact of pilot contamination, for instance by proposing greedy-based pilot assignment methods [102, 103]. A graph coloring-based pilot allocation scheme is also proposed in [104] to reduce the impact of pilot contamination assuming that only a limited number of APs serves each user. A different approach is taken in [105] by focusing on reducing the pilot overhead through joint channel estimation and data detection, which is formulated as a biconvex optimization problem and solved using a forward-backward splitting algorithm.

As evidenced by the above works, progress has been made in developing channel estimation techniques to enable 6G systems. Yet there are still open problems and more effort needs to be expended to find the most optimal solutions. It is expected that future solutions will continue to build on the sparsity properties of the channel matrix and the power of deep learning to produce algorithms that combine high spectral efficiency with low computational complexity and high estimation accuracy. Only few works have considered the impact of various RF impairments in channel estimation thus far [106], although these impairments are expected to affect the performance of 6G systems. Furthermore, 6G systems support high levels of mobility, while most of the developed works focus on stationary channels.

## Non-Orthogonal Multiple Access

As 6G systems are expected to support extremely high data rates for numerous users and devices, orthogonal multiple access (OMA) schemes, which have been the mainstay of previous generations of wireless systems, may not be able to cope with the increasing demand, resulting in undesired limitations on the improvement in spectral efficiency. Non-orthogonal multiple access is a promising technology that can effectively solve this problem. At the cost of increased receiver complexity, NOMA allows multiple users to use the same time/frequency resources, separating them in power or code domains [107]. The most common form is power-domain NOMA, which multiplexes users by superposing them at the transmitter side using different power levels. At the receiver side, successive interference cancellation (SIC) is used to recover the transmissions of the different users. NOMA exhibits both improved throughout and fairness in comparison to OMA, and it is expected to play a key role in 6G systems.

**Figure 6:**
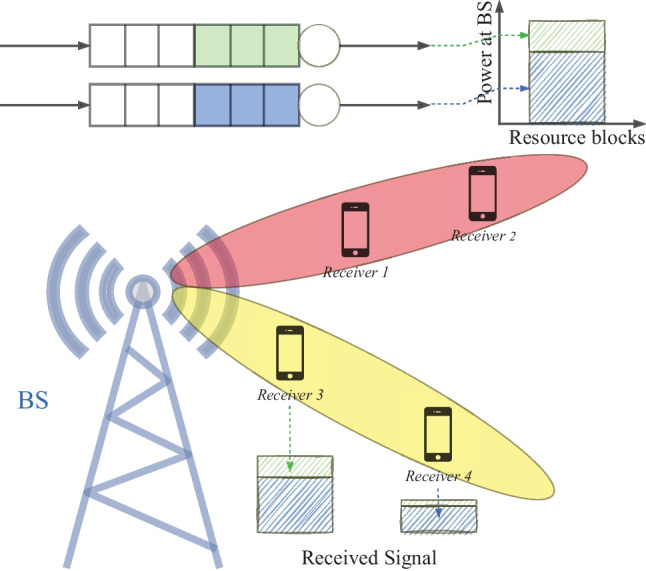
NOMA-MIMO scheme.

The integration of NOMA and MIMO technologies has been highlighted as a powerful approach to achieving high spectral efficiency and better wireless services [108] (Fig. 6). In contrast to SISO NOMA, where the focus is to optimize power allocation among users, MIMO-NOMA provides additional degrees of freedom through beamforming in the spatial domain. As noted in [107], the beamforming and SIC problems become coupled in the MIMO-NOMA setup, since the design of the beamformer has a direct impact on both the signal power and the interference power of the different users. Since the SIC performance of MIMO-NOMA largely depends on the decoding order of the users, it needs to be designed jointly with the beamformer, which opens the way to a new class of joint optimization problems.

A single-cluster MIMO-NOMA system is investigated in [109], where the authors aim to optimize the power allocation and beamforming in order to maximize the sum rate of single-antenna users for a given SIC order. The optimization considers both a total transmit power constraint as well as an additional constraint to protect weak users. The resulting problem is non-convex and is solved through a successive convex optimization approach based on minorization-maximization. The simulation results indicate that MIMO-NOMA may be superior to traditional Zero-Forcing (ZF) beamforming when the number of users is significantly higher than the number of transmit antennas at the BS. A two-user downlink MIMO-NOMA system is considered in [110], where the ergodic capacity is maximized for a given decoding order based on statistical CSI and optimizing the transmit covariance matrix. Inspired by the H-BLAST scheme, a MIMO-NOMA system with layered transmission is proposed in [111], and the power allocation is optimized to maximize the sumrate. Furthermore, the authors of [112] identify a “quasi-degraded” channel condition for the two-user MISO channel, and accordingly optimize the beamforming for MISO-NOMA by minimizing the transmit power under user rate constraints.

In the above scenarios, all the users are grouped into the same cluster, and hence each user interferes with all other users in the network. It can be prohibitively complex to optimize both the beamformer and the decoding order in such cases, especially when the number of users is large. To overcome this problem, multi-cluster MIMO-NOMA is proposed in [113], where each cluster consists of several users that share the same beamformer. This allows grouping users with similar spatial characteristics into the same cluser to minimize inter-cluster interference. Furthermore, it is sufficient to perform SIC only for users within the same cluster, which reduces the decoding complexity. Using ZF beamforming to eliminate inter-cluster interference, the authors in [113] develop clustering algorithms to maximize the fairness for downlink MIMO-NOMA. The authors of [114] propose a general MIMO-NOMA framework applicable to both uplink and downlinke transmission, by employing the concept of signal alignment. Using signal alignment, the multi-user MIMO-NOMA scenario is decomposed into several single-antenna NOMA channels. Both fixed power allocation and cognitive-radio inspired power allocation are considered. A precoding/detection vector selection scheme is also developed in order to efficiently exploit the available degrees of freedom.

A two-stage beamforming scheme is proposed for two-user downlink MISO-NOMA in [115] where the first stage eliminates the inter-cluster interference through ZF beamforming, while the second stage employs intra-cluster beamformers to minimize the transmit power. The authors of [116] consider a beamforming design for downlink MIMO-NOMA to cancel a substantial part of the inter-cluster interference when the number of transmit antennas of the BS is smaller than the total number of user antennas. User clustering is also considered in [116], where a method is proposed that assigns users with maximally distinct channel gains to each cluster in order to optimize SIC performance. The beamformer optimization problem under imperfect CSI is investigated in [117] using successive convex optimization and semi-definite programming.

The previous works investigate the combination of NOMA with conventional MIMO. As massive MIMO is expected to play a leading role in 6G systems, massive MIMO-NOMA is considered an attractive research area, given the large number of spatial degrees of freedom available at the BS. Massive MIMO has traditionally focused on underloaded systems where the number of users is smaller than the number of BS antennas. Hence, the spatial degrees of freedom provided by massive MIMO may not be enough to efficiently handle overloaded systems with an excessive number of users. The power domain multiplexing enabled by NOMA can facilitate serving more users. The authors of [118] consider the overloaded massive MIMO-NOMA scenario where the number of users is larger than the number of antennas, and propose a Gaussian message passing (GMP) multiuser detection scheme. The GMP exhibits a complexity that is linear in the number of users. A user clustering scheme is proposed for cell-free massive MIMO-NOMA in [119] and the resulting sum rates are derived considering intra-cluster pilot contamination, inter-cluster interference and imperfect SIC.

As massive MIMO-NOMA requires accurate CSI to realize its potential, the authors of [120] consider two pilot schemes, one of orthogonal pilots, and the other where pilots are superimposed with the data. A data-aided channel estimation scheme is investigated, where partially decoded data are used to improve channel estimation. The use of NOMA is shown to mitigate the impact of pilot contamination. Channel estimation for uplink massive MIMO-NOMA is also studied in [121], using semi-blind estimation strategies. Group successive interference cancellation is employed in conjunction with semi-blind estimation in a multi-cell scenario. After dividing the users into multiple groups according to their large scale fading, eigenvalue decomposition is applied to separate the signal subspaces of different groups using the same pilot sequences. The proposed method is shown to outperform conventional estimation techniques. The authors of [122] propose a method to alleviate the impact of channel estimation and SIC imperfections by employing a successive sub-array activation (SSAA) diversity scheme, resulting in better performance.

The application of NOMA at mmWave and THz bands is another avenue to combine high data rates with increased connectivity. As noted in [107], the number of users that can be supported at such high frequencies is limited by the number of available RF chains. NOMA can resolve this limitation by increasing the number of users through power domain multiplexing. The authors of [123] propose the integration of NOMA with beamspace MIMO systems operating in mmWave settings. A ZF precoding scheme is also developed to reduce the inter-beam interference, while a dynamic power allocation scheme is developed to optimize the sum rate, which considers both inter-beam and intra-beam power allocation. The proposed system is shown to provide superior energy and spectrum efficiency compared to systems that do not utilize NOMA. To guarantee the rate performance for all users,the authors of [124] maximize the minimal rate of the system using max-min fairness, assuming that NOMA users in the same beam share the same precoding vector. The minimal rate maximization problem is non-convex due to the inter-beam and intra-beam interferences. Hence, alternating optimization is used to solve the power allocation and precoding problems. The integration of simultaneous wireless information and power transfer (SWIPT) with mmWave massive MIMO-NOMA is proposed in [125], where hybrid precoding is considered to reduce the number of RF chains. A power splitting receiver is proposed to allow each user to extract both information and energy. Joint optimization of the user power allocation and power splitting factors for SWIPT is solved through an iterative optimization algorithm. Furthermore, a novel cluster grouping scheme is proposed in [126] to reduce the inter-cluster interference for mmWave MIMO-NOMA with hybrid precoding. MIMO-NOMA using the THz band is studied in [127], where user clustering, hybrid precoding and power allocation are optimized to maximize energy efficiency.

The integration of RIS and NOMA technologies is also considered an attractive option since the careful deployment and selection of reflection coefficients of RISs can increase the channel disparity among users, which would lead to higher NOMA gains [107]. Furthermore, this integration can aid in satisfying the QoS constraints of users, since the QoS constraints are not necessarily of the same order as the decoding order, which is dictated by channel conditions. The ability to modify the channels through RIS can help in satisfying the constraints. Hence, there has been a significant interest in combining the two technologies. The work in [128] considers a downlink MISO RIS-aided NOMA system, where the active beamforming of the BS and the passive beamforming of the RIS are jointly optimized to maximize the sum rate of all users subject to various constraints. Alternating optimization is used to solve the resulting non-convex optimization problems. Furthermore, low-complexity user ordering schemes are proposed in [129], which achieve close performance to the exhaustive search used in [128]. A signal cancellation design is developed in [130], where the reflection coefficients are selected to reduce inter-cluster interference of the different NOMA clusters. The signal cancellation enabled by RIS relaxes the constraints on the number of transmit and receive antennas.

Given the growing appreciation for the power of deep learning, it has also been applied to solve various NOMA-related research problems. Deep learning is used to acquire end-to-end CSI in [131]. In particular, a long short-term memory (LSTM) network is integrated into the NOMA system, to obtain channel characteristics automatically. User clustering through deep learning is proposed in [132]. A deep learning framework is proposed in [133] to maximize the sum rate and energy efficiency for MIMO-NOMA. A deep convolutional neural network, aided by training algorithms is used to address the power allocation problem. Deep learning is used in [134] to maximize the sum rate for a downlink NOMA system by optimizing the power allocation.

It is obvious form the above works that NOMA will play an important role in the evoluation of 6G systems due to its attractive spectral efficiency. Furthermore, NOMA can be effectively integrated with other emerging technologies such as massive MIMO, mmWave communication, and RIS. NOMA also lends itself to the application of machine learning and deep learning techniques to improve performance and/or reduce computational complexity. Important challenges remain to be addressed, however. While most works assume perfect channel knowledge, accurate channel estimation requires a significant training overhead, which may have a non-negligible impact on spectral efficiency. Furthermore, while most works consider perfect SIC, error propagation remains a importance consideration in practical SIC for NOMA systems [107]. Finally, the efficient design of modulation and detection schemes for NOMA remains an open problem since most works base their analysis on the ideal Gaussian signaling [135].

## Optical Wireless Communications

Optical wireless communications (OWC) is an efficient and mature technology that has been developed alongside cellular technology, which has only used radio spectrum. OWC can potentially satisfy the demanding requirements at the backhaul and access network levels beyond 5G networks. As the 6G development gains momentum, comprehensive research activities are being carried out on developing OWC-based solutions capable of delivering ubiquitous, ultra-high-speed, low-power consumption, highly secure, and low-cost wireless access in diverse application scenarios [136, 137]. In particular, this includes using hybrid networks that combine OWC with radio frequency or wired/fiber-based technologies. Solutions for IoT connectivity in smart environments are being investigated for developing flexible and efficient backhaul/fronthaul OWC links with low latency and support for access traffic growth [138].

The OWC technology covers the three optical bands of infrared (IR: 187-400 THz, 750-1600 nm wavelength), visible light (VL: 400-770 THz, 390-750 nm) and ultraviolet (UV: 1000-1500 THz, 200-280 nm). Free space optics (FSO) and visible light communications (VLC) are commonly used terms to describe various forms of OWC technology [139]. FSO mainly refers to the use of long-range, high-speed point-to-point outdoor/space laser links in the IR band [140], while VLC relies on LEDs operating in the VL band, mostly in indoor vehicular environments [141].

**Figure 7:**
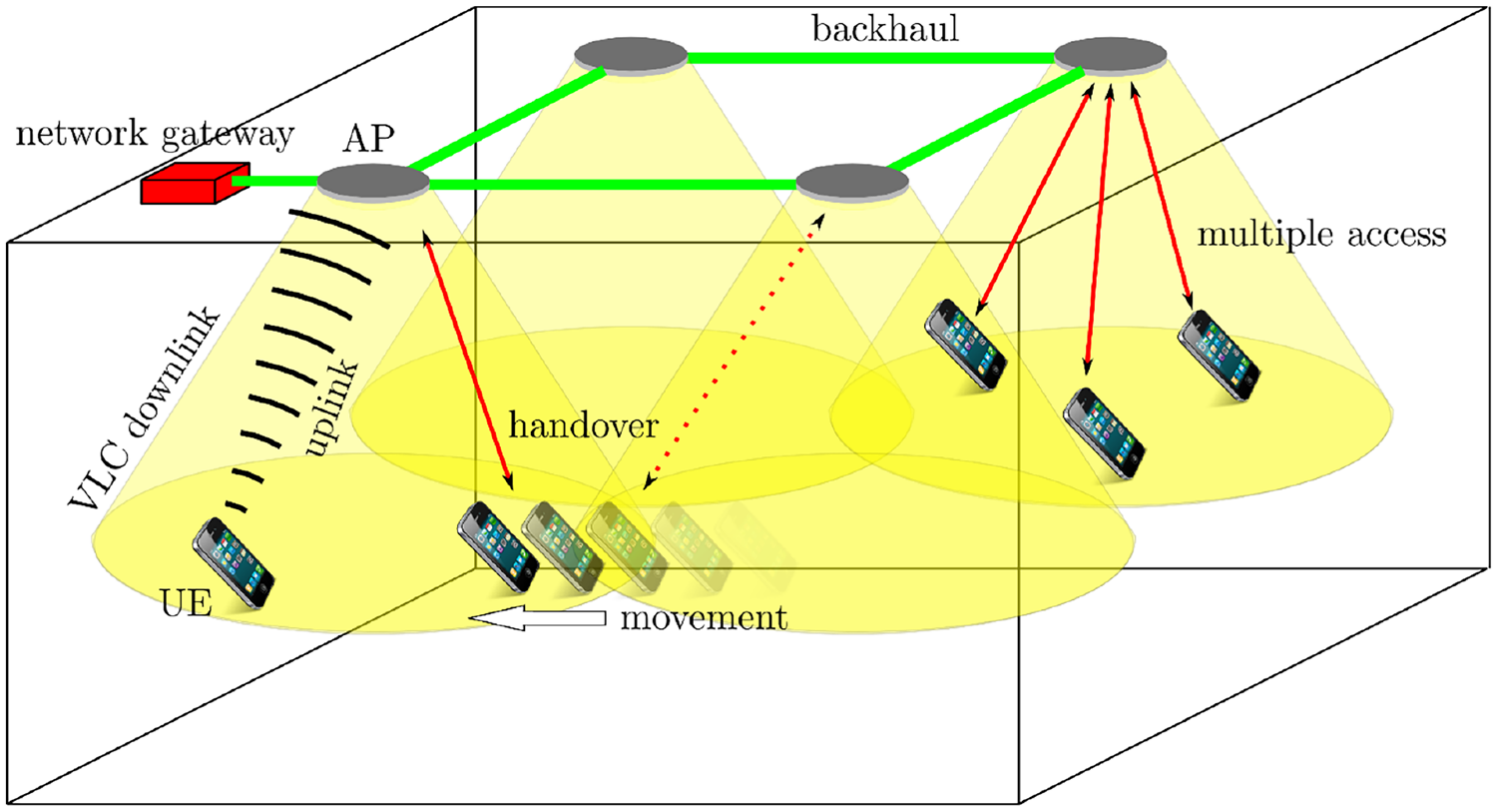
Bi-directional, point-to-point and mobile communications with networked wireless access.

In comparison to RF, OWC systems offer significant technical and operational advantages including, but not limited to **i)** huge bandwidth, which leads to high data rates; e.g., a recent FSO system achieved a world record data rate of 13.16 Tbps over a distance of 10 km [139], and multiple Gbps in indoor VLC setups [142]; **ii)** operation in the unregulated spectrum, thus no license fees and associated costs; **iii)** immunity to the RF electromagnetic interference; **iv)** a high degree of spatial confinement, offering virtually unlimited frequency reuse capability, inherent security at the physical layer, and no interference with other devices; **v)** a green technology with high energy efficiency due to low power consumption and reduced interference. With such features, OWC is well-positioned to be a prevailing complement to RF wireless solutions from micro- to macro-scale applications, including intra/inter-chip connections and indoor wireless access (WA) localization, underwater, outdoor and space point-to-point links, etc. Beyond the state-of-the-art, however, the dominance of RF-based WA technologies will be challenged. LiFi [142] is a promising technology to provide local broadband connectivity [141]. As shown in Fig. 7, VLC provides high-speed, bi-directional, networked data delivery through the lighting infrastructure. When a device moves out of the light cone of one light source, the services can be handed over to the next light source, or eventually, the device can be connected and handed over to an RF-based system if optical access is no longer provided. In VLC, all the baseband signal processing at the transmitter and the receiver is performed in the electrical domain, and intensity modulation/direct detection is the most practical scheme. LEDs with large FoV or laser diodes with a small FoV encode and transmit data over the line-of-sight (LOS)/NLOS optical channel. Photo-detectors at the receiver convert data, carrying light intensity back to electrical signals for baseband processing. A VLC-enabled device inside a pocket or briefcase cannot be connected optically, which is one example of why a hybrid optical-radio wireless network is needed. A reconfigurable optical-radio network is a high performance and highly flexible communications system that can be adapted for changing situations and different scenarios [143].

Performance-wise, data throughput below 100 Mbps can be achieved with relatively simple optical transceivers and off-the-shelf components. Data rates of up to hundreds of Gbps have been demonstrated in laboratory conditions, and it is expected that even Tbps-communications will be achieved in the future.

Open research directions in OWC and in VLC toward 6G include:

- Accurate VLC channel modeling and characterization for various deployment scenarios with a particular emphasis on user-dense environments. Incorporating user mobility and device orientation into the VLC channel models and combining VLC and RF systems [144, 145].
- New non-coherent physical-layer transmission schemes such as spatial modulation and its variations can be used, as well as non-orthogonal communication schemes such as MIMO [144, 145].
- Exploiting R-G-B LEDs, developing new materials and optoelectronic devices (e.g., fast non phosphorous LEDs, micro-LEDs), very fast switching mechanisms between optical and radio systems, etc. [146].
- Use of OWC to provide secure and safe connectivity in in-body communications applications, including communications to and from the body [147], communications between sensors inside the body, etc. Recent results have shown that near-infrared light can be used for this purpose [148, 149].
- Design of new and novel optical IoT, new devices, and interfaces to tackle the efficient generation, routing, detection, and processing of optical signals [150].
- For ultra-dense IoT scenarios, there are a number of open challenges that appeal for a radical rethinking of network topologies and the design of media access control and network layers in OWC [151].
- In VLC, to account for multi-user scenarios and user mobility, robust low-complexity multiple access techniques need to be designed, together with efficient cellular architectures with user-scheduling and intra-room handover capability, achieving high capacity, low latency, and fairness [138, 152].
- At the MAC layer, robust link quality estimators will be developed due to the small packet sizes used in machine-to-machine (M2M) applications and constraints on sensor devices. Routing algorithms will be devised taking into account the optimal trade-off between the link capacity, connectivity, latency, and energy consumption [141, 153, 154].
- In medium-range OWC, the effects of weather and environmental conditions, ambient noise, and link misalignment need to be investigated to enable connectivity between distant vehicles. Physical-layer designs need to be built upon multi-hop transmission to reduce the delay in transmission of road safety-related information [138, 141, 154].
- For long-range links, extensive research should be carried out to minimize the terminal size to enable the technology to be integrated into small satellites, e.g., CubeSats, with data rates up to 10 Gbps and for the investigation of how to deal with cloud obstruction. Site diversity techniques and smart route selection algorithms should be devised for satellite links and airborne networks, respectively. Also, hybrid RF/FSO and optimized multi-hop transmission techniques should be investigated to improve link reliability between satellites or high altitude platforms (HAPs) [155, 156].

## Physical-Layer Security

### PLS Through Wireless Communications

Given the intrinsic nature of future 6G services, with the increase in traffic volumes over wireless networks, data privacy and security are a predominant concern for users and network administrators. Providing wireless networks both in RF and optical domains with trusted communications is a crucial objective for successfully deploying services, such as perpetual data upload and download, caching, and inter-networking. Hence, security should be considered an essential performance requirement in 6G systems, and signal processing can strongly support it.

Physical layer security (PLS) can play a vital role in enhancing cyber-security in 6G wireless networks. It refers to transmission schemes that exploit dissimilarities among the channels of different receivers to hide information from unauthorized users without reliance on upper network layer encryption techniques. The secrecy capacity is used as a performance measure to determine the maximum communication rate that guarantees the authorized receiver’s reliable reception of the secret message. PLS mechanisms, that are mainly based on advanced signal processing techniques, will also help reduce the latency and the complexity of novel security algorithms.

Two well-known PLS techniques are based on either applying beamforming in the direction of the legitimate user or generating a friendly jamming signal that creates an artificial noise, which lies in the null space of the legitimate user. After combining the confidential information with the jamming signal at the transmitter side, only the eavesdropper will experience destructive effects from the jamming signal [157–159]. These techniques are anyway based on having knowledge of the location of the eavesdropper or at least an estimation of its channel state information, which is often hard to get. In [160, 161] a noise-loop modulation is proposed, which uses equipment noise to modulate the information bits to provide confidentiality without any knowledge about the eavesdropper.

### PLS Through Optical Wireless Communications

**Figure 8:**
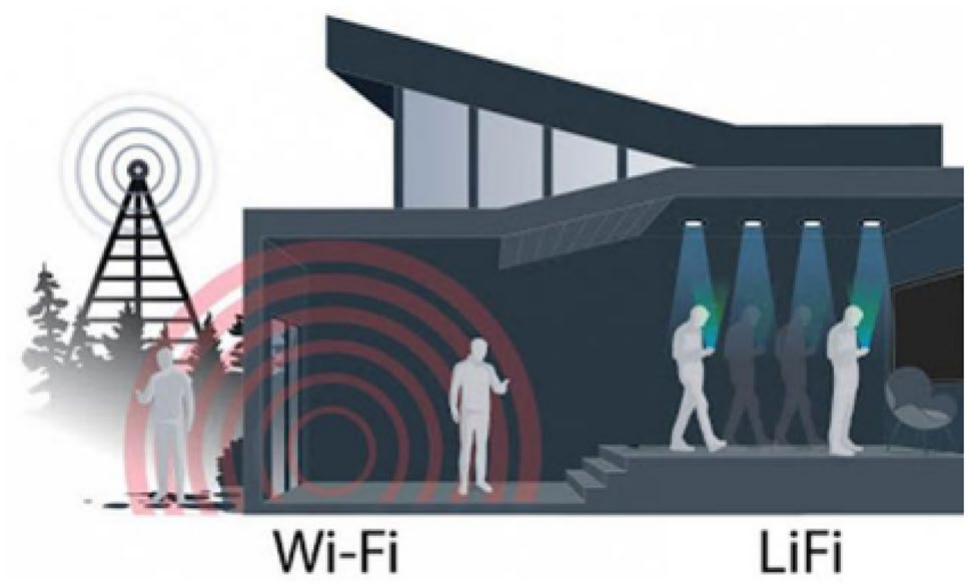
WiFi-LiFi hybrid communications.

In VLC, PLS is especially important when a large physical indoor environment is accessible to or shared by multiple users and potential eavesdroppers. Some examples include meeting rooms, public libraries, airplanes, hospitals, etc. Light does not propagate through opaque objects (e.g. walls), it is directional and light beams can be formed with signal processing efforts. It is, therefore, possible to significantly reduce the possibilities of man-in-middle attacks in LiFi compared to WiFi (Fig. 8). It has been shown that the secrecy capacity of a LiFi network is 20 times higher than that of a WiFi network.

PLS methods employing signal processing techniques in MIMO-VLC have been proposed recently. In MIMO-based index-modulation (IM) techniques [157, 162], a random switching among the antennas (LEDs) is exploited to generate a strong and friendly jamming signal, which is invaluable for PLS applications. In precoding approaches [163–165], the channel state information at the transmitter (CSIT) of the legitimate user is used to construct the precoding matrix coefficients so that the confidential message is perceived by the legitimate user clearly while the eavesdropper’s bit error rate (BER) performance is degraded substantially.

## Research Directions and Challenges

In addition to the core signal processing technologies described in the previous section, there are several emerging technologies possibly having a significant impact on 6G systems. A few possible ones are briefly discussed below.

### Machine Learning for 6G Design

The availability of a large amount of data, advanced technological progress, the revolution in optimization tools, the availability of powerful processing units, a huge amount of available memory, and systematic data mining and extraction techniques may jointly lead the basis to the AI utilization to achieve further improvements to the physical, MAC, and network layers in B5G networks. However, the available resources and the technological development remain limited, hence, the ML paradigms look promising in 6G as the communication performance requirements keep aggressively increasing. Although a plethora of machine learning (ML) based 5G can be seen in literature, the concepts are taken from mature technologies, such as computer vision and natural language processing domains, and exploited in communication systems, and hence, many challenges are raised such as the selection of optimal data representations, loss-functions, and training strategies. In other words, 6G should have its own definitions, algorithms, techniques, and tools of ML for wireless communication scenarios. The training complexity and the generalization capabilities of the trained models in wireless communications are really challenges. Most existing models are not adaptable to changes in channel statistics, realizations, and modulation orders. In addition, there is a lack of datasets to benchmark and compare the performance of ML models and algorithms [166]. Training at wide ranges of SNR should be considered as it severely impacts training time. Moreover, wireless communication systems usually deal with complex baseband representation while the most used neural networks (NN) utilize real arithmetic. Therefore, to meet the requirements of 6G, there is a need for NNs to operate with complex numbers. ML could be exploited at the MAC layer to have an adaptive control channel based on the traffic and other requirements of the connected devices. ML also could be exploited at the network layer to develop routing protocols.

### Integrated Communications and Sensing

6G is envisioned to continue to transform from connected people and connected things, to connected “intelligences”. The mobile network will connect large numbers of intelligent devices, equipped with not only communication but also sensing capabilities at the same time. These intelligent nodes will have the capability to sense their surroundings, and exchange their observations through communication: the cognitive circle (sense-communicate-think-act) applied to intelligent networks. AI can be combined with sensing capability such that the network will have human-like cognition capabilities [167]. Communications and sensing can be integrated and work jointly to give benefits to each other [168]. When those are integrated to a single device to coexist based on different system designs and waveforms (possibly operating on separate frequency bands), the term ICAS is often used. When the systems and waveforms are jointly designed to serve both functionalities, the solution is often called *joint communications and sensing* (JCS). Both approaches can provide the system with accurate localization, imaging, and, in general, high-resolution environment map. Those can improve communication performance, opening the way to a broad range of new services [169].

The use of higher frequency spectrum enables a high-accuracy localization and tracking, together with the capability of imaging and 3D mapping of the environment where communication subjects (humans, machines, things) are immersed. Similarly, an accurate sensing capability in/on the human body could open the way to augmented human communications and very accurate gesture/activity recognition [170].

In such scenario, the role of signal processing is crucial to model the environment and adapt the communication: from one side the model-based methods (geometric optics, statistical signal processing, optimization theory, etc.) [171] and, from the other side, model-free methods (data-driven ML and AI) [172]. Although the former methods are rigorous and provide certain performance and design optimization, the latter can be used when the modelling is too complex to be obtained or to quickly adapt to changes.

### Internet of Bio-Nano-Things

**Figure 9:**
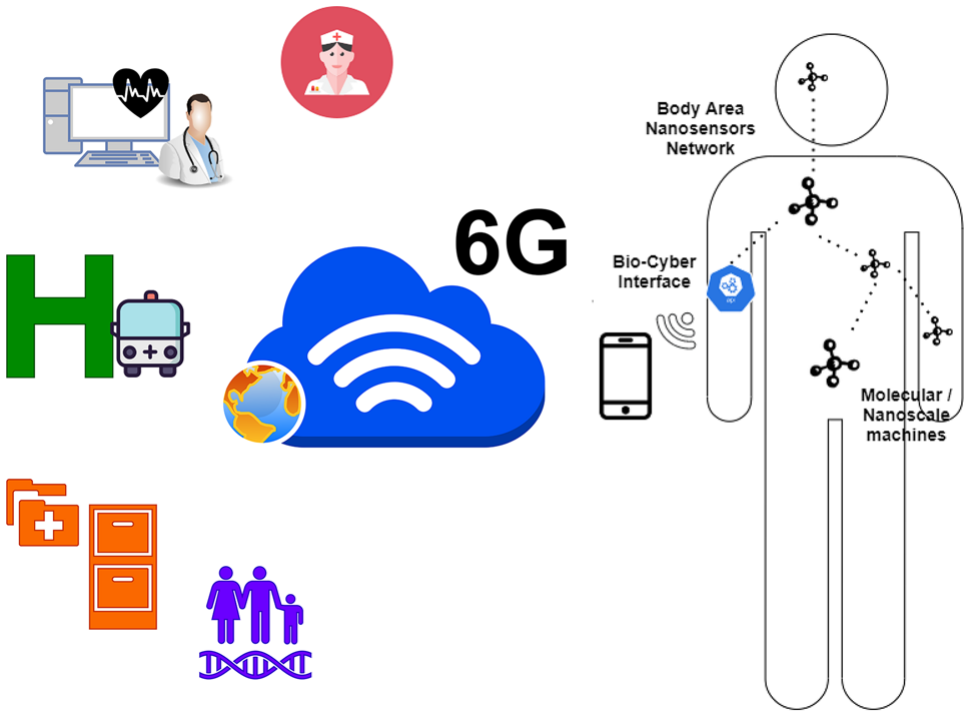
In-body bio nano networks as part of the Net.

The Internet of Things (IoT) is one of the most important element of 6G systems. Things refer to interconnected machines and objects with embedded computing capabilities employed to extend the Internet to many application domains such as health, home, office, transportation, food, space, ocean, etc. 6G should be the connectivity “tissue” that makes all these different resourced and sized devices to connect each other. There are many application domains where Things are required to be tiny, concealable, and non-intrusive. Nanomaterials (graphene, etc.) have stimulated the recent concept of Internet of NanoThings (IoNT), referring to interconnection of nano-scale (artificial) devices. More recently bio-nano-devices have been investigated, as a potential disrupting method to take and deliver information inside the human body. The concept of Internet of Bio-Nano Things (IoBNT) was firstly introduced in [173], envisioning the use of natural or engineered bio-devices with embedded computing and communication capabilities (by using bio-chemical signals). 6G networks should support the exchange of information from inside the body to Internet (Fig. 9), following the concept that the human body will be part of the global Network [174]. Bio-Nano-Things, together with the 6G support, could enable applications such as intra-body sensing and actuation systems for a new era of health monitoring and treatment [175].

## Summary and Conclusions

In this paper an overview of the signal processing techniques for future 6G networks is provided. The signal processing chain is considered as a whole, from the conditioning of the signal at transmission to MIMO detection, from channel coding to channel estimation, from multicarrier (radio) modulation to optical wireless communications. Physical-layer security and bio-nano in-body communications are also considered as an important part of next generation networks.

DPD in the THz band large array transmitters is a significant challenge together with the design of efficient transmit and receive processing chains providing energy and power efficient solutions given the large numbers of ADCs and digital-to-analog converters. Channel estimation is challenging also due to the rapid channel variations and phase noise in the THz band. The large antenna arrays and the introduction of RIS technology further complicates the processing. Efficient channel coding and practical implementation of Tb/s decoders is by no means trivial and requires both algorithmic and architectural innovations. The multicarrier OFDM based air interfaces and the design of NOMA solutions together with realistic transceivers is an important challenge determining to large extent the power and energy efficiency characteristics of the emerging 6G networks. OWC is a promising solution for special indoor use cases providing inherent security and avoiding the electromagnetic interference problems typical for the microwave and THz bands. PLS may also solve elegantly some of the security and privacy concerns, which are very significant in the data avalanche enabled in part by 5G and even more by 6G.

All the above mentioned signal processing challenges still require significant research efforts. In addition, some more future directions were also identified and discussed. There is a room for fundamental research to exploit the artificial intelligence and deep learning in the design of DPD, precoders and detectors. What is more, ML is expected to play some role in the design of actual air interfaces, modulation, waveforms, channel decoders etc. The benefit of such an approach is still debatable and more research is necessary. The merger of communications and sensing will be one key feature of 6G and AI/ML will play a role therein, too. The internet of bio-nano-things was also discussed as one potential direction for future networks. It may, however, be more a technology for 7G than 6G. Wireless quantum computing and communications is also an important emerging area, which may also mature for practical realization in the 7G systems expected to emerge in 2040s.

## Data Availability

No data or other material was produced in this study.
